# 基于测序技术的核糖核酸修饰定位分析方法研究进展

**DOI:** 10.3724/SP.J.1123.2023.12025

**Published:** 2024-07-08

**Authors:** Jun XIONG, Tian FENG, Bi-Feng YUAN

**Affiliations:** 武汉大学公共卫生学院,湖北 武汉 430071; School of Public Health, Wuhan University, Wuhan 430071, China

**Keywords:** 表观转录组学, 核糖核酸修饰, 定位分析, 高通量测序, 单碱基分辨率, epitranscriptome, RNA modification, mapping analysis, high-throughput sequencing, single-base resolution

## Abstract

迄今为止,已在核糖核酸(RNA)中发现了170多种不同的化学修饰,这些修饰分布在各种类型的RNA中,包括信使RNA(mRNA)、核糖体RNA(rRNA)、转运RNA(tRNA)和小核RNA(snRNA)等。RNA修饰在广泛的生物过程中发挥着至关重要的作用,如调节基因表达、维持RNA稳定性和促进蛋白质翻译等。RNA修饰构成了基因表达调控的新层面,即“表观转录组”。RNA修饰以及相关修饰酶(writer)、去修饰酶(eraser)和修饰识别蛋白(reader)的发现,为研究RNA修饰的动态调控及生理功能提供了重要依据。随着RNA修饰检测技术的发展,RNA表观转录组学研究进入了单碱基分辨率、多层面、全覆盖的发展阶段。这些覆盖全转录组的分析方法有助于发现新的RNA修饰位点,对于阐明表观转录组学的分子调控机制、探索RNA修饰的疾病关联及临床应用具有重要意义。根据处理方式及测序原理的差异,现有的RNA修饰测序技术可以分为直接高通量测序、抗体富集测序、酶辅助测序、化学辅助测序、代谢标记测序和纳米孔测序。这些方法伴随着RNA修饰功能的研究大大拓展了人们对表观转录组学的认识。在这篇综述中,我们总结了近年来有关RNA修饰检测技术的进展,重点聚焦于不同方法的基本原理、优势和局限性。针对不同类型的RNA修饰,探讨测序分析方法,对未来新型RNA修饰定位技术的开发具有指导意义,有助于在全转录组范围内研究RNA修饰的功能。

## Abstract (en)

Over 170 chemical modifications have been discovered in various types of ribonucleic acids (RNAs), including messenger RNA (mRNA), ribosomal RNA (rRNA), transfer RNA (tRNA), and small nuclear RNA (snRNA). These RNA modifications play crucial roles in a wide range of biological processes such as gene expression regulation, RNA stability maintenance, and protein translation. RNA modifications represent a new dimension of gene expression regulation known as the “epitranscriptome”. The discovery of RNA modifications and the relevant writers, erasers, and readers provides an important basis for studies on the dynamic regulation and physiological functions of RNA modifications. Owing to the development of detection technologies for RNA modifications, studies on RNA epitranscriptomes have progressed to the single-base resolution, multilayer, and full-coverage stage. Transcriptome-wide methods help discover new RNA modification sites and are of great importance for elucidating the molecular regulatory mechanisms of epitranscriptomics, exploring the disease associations of RNA modifications, and understanding their clinical applications. The existing RNA modification sequencing technologies can be categorized according to the pretreatment approach and sequencing principle as direct high-throughput sequencing, antibody-enrichment sequencing, enzyme-assisted sequencing, chemical labeling-assisted sequencing, metabolic labeling sequencing, and nanopore sequencing technologies. These methods, as well as studies on the functions of RNA modifications, have greatly expanded our understanding of epitranscriptomics. In this review, we summarize the recent progress in RNA modification detection technologies, focusing on the basic principles, advantages, and limitations of different methods. Direct high-throughput sequencing methods do not require complex RNA pretreatment and allow for the mapping of RNA modifications using conventional RNA sequencing methods. However, only a few RNA modifications can be analyzed by high-throughput sequencing. Antibody enrichment followed by high-throughput sequencing has emerged as a crucial approach for mapping RNA modifications, significantly advancing the understanding of RNA modifications and their regulatory functions in different species. However, the resolution of antibody-enrichment sequencing is limited to approximately 100-200 bp. Although chemical crosslinking techniques can achieve single-base resolution, these methods are often complex, and the specificity of the antibodies used in these methods has raised concerns. In particular, the issue of off-target binding by the antibodies requires urgent attention. Enzyme-assisted sequencing has improved the accuracy of the localization analysis of RNA modifications and enables stoichiometric detection with single-base resolution. However, the enzymes used in this technique show poor reactivity, specificity, and sequence preference. Chemical labeling sequencing has become a widely used approach for profiling RNA modifications, particularly by altering reverse transcription (RT) signatures such as RT stops, misincorporations, and deletions. Chemical-assisted sequencing provides a sequence-independent RNA modification detection strategy that enables the localization of multiple RNA modifications. Additionally, when combined with the biotin-streptavidin affinity method, low-abundance RNA modifications can be enriched and detected. Nevertheless, the specificity of many chemical reactions remains problematic, and the development of specific reaction probes for particular modifications should continue in the future to achieve the precise localization of RNA modifications. As an indirect localization method, metabolic labeling sequencing specifically localizes the sites at which modifying enzymes act, which is of great significance in the study of RNA modification functions. However, this method is limited by the intracellular labeling of RNA and cannot be applied to biological samples such as clinical tissues and blood samples. Nanopore sequencing is a direct RNA-sequencing method that does not require RT or the polymerase chain reaction (PCR). However, challenges in analyzing the data obtained from nanopore sequencing, such as the high rate of false positives, must be resolved. Discussing sequencing analysis methods for various types of RNA modifications is instructive for the future development of novel RNA modification mapping technologies, and will aid studies on the functions of RNA modifications across the entire transcriptome.

核酸表观遗传学是指不改变核酸(DNA和RNA)序列的情况下,通过化学修饰或其他方式引起的遗传信息的变化^[1]^。这些变化可以影响基因的表达和功能,并且在某些情况下能够遗传给后代^[2]^。核酸表观遗传学的研究领域主要集中在DNA和RNA修饰上,这些修饰在细胞分化、发育、疾病以及对环境变化的响应中起着关键作用^[3⇓⇓⇓⇓⇓-9]^。RNA分子由腺嘌呤(A)、鸟嘌呤(G)、胞嘧啶(C)和尿嘧啶(U)4种碱基组成,除此之外,RNA中还存在修饰碱基^[10]^。

迄今为止,已在生命的三界系统中发现了170多种不同化学结构的RNA修饰^[11⇓⇓⇓⇓⇓-17]^。RNA修饰存在于不同类型的RNA中,如信使RNA(messenger RNA, mRNA)、核糖体RNA(ribosomal RNA, rRNA)、转运RNA(transfer RNA, tRNA)和小核RNA等^[18⇓⇓⇓⇓⇓⇓-25]^。RNA修饰能够通过结合蛋白、修饰酶、去修饰酶和抑制蛋白等调控基因表达,并在许多生理和病理过程中发挥功能^[26⇓⇓⇓⇓-31]^。

检测技术的发展推动了核酸修饰功能的研究^[7,32⇓⇓⇓⇓⇓⇓⇓⇓⇓-42]^。以mRNA为例,迄今为止,已在mRNA中发现了10多种RNA修饰^[43⇓⇓-46]^,包括*N*^6^-甲基腺苷(*N*^6^-methyladenosine, m^6^A)、*N*^1^-甲基腺苷(*N*^1^-methyladenosine, m^1^A)、*N*^6^,2*'-O*-二甲基腺苷(*N*^6^,2*'-O*-dimethyladenosine, m^6^Am)、肌苷(inosine, Ino)、7-甲基鸟苷(7-methylguanosine, m^7^G)、5-甲基胞苷(5-methylcytidine, m^5^C)、*N*^4^-乙酰胞苷(*N*^4^-acetylcytidine, ac^4^C)、假尿苷(pseudouridine, Ψ)、5-甲基尿苷(5-methyluridine, m^5^U)、二氢尿苷(dihydrouridine, D)和2'-*O*-甲基化(2'-*O*-methylation, Nm)(见图1)。作为哺乳动物体内最丰富的内部mRNA修饰之一,m^6^A在RNA的稳定性、定位、剪接和翻译等多种生物过程中发挥着重要作用,能够通过染色体相关调控RNA(carRNA)影响染色质状态和转录过程,与癌症发生密切相关^[47⇓⇓-50]^。mRNA中,m^1^A能够促进翻译,tRNA的m^1^A58修饰是一个保守位点,能够影响翻译启动和tRNA的稳定性并与肝癌发生等密切相关^[51⇓⇓⇓⇓-56]^。m^7^G修饰不仅存在于mRNA的5'-帽子,在mRNA的内部也广泛存在并影响翻译和对环境压力的调节^[57⇓-59]^。Ino是哺乳动物细胞RNA编辑功能的重要修饰,与密码子突变、可变剪接、RNA识别蛋白调控及miRNA介导的基因沉默等功能密切相关^[60⇓-62]^。Ψ普遍存在于各类RNA中,tRNA和rRNA中的Ψ能够稳定RNA的二级结构。在mRNA中,Ψ能够促进翻译,并促进氨基酸的异常替换^[63⇓-65]^。Nm是最常见的RNA修饰之一,在几乎所有RNA中都普遍存在。在真核生物mRNA中,Nm与5'-帽子形成、翻译和mRNA稳定性密切相关,在病毒抗免疫反应中起着重要作用^[61,66⇓-68]^。

**图1:**
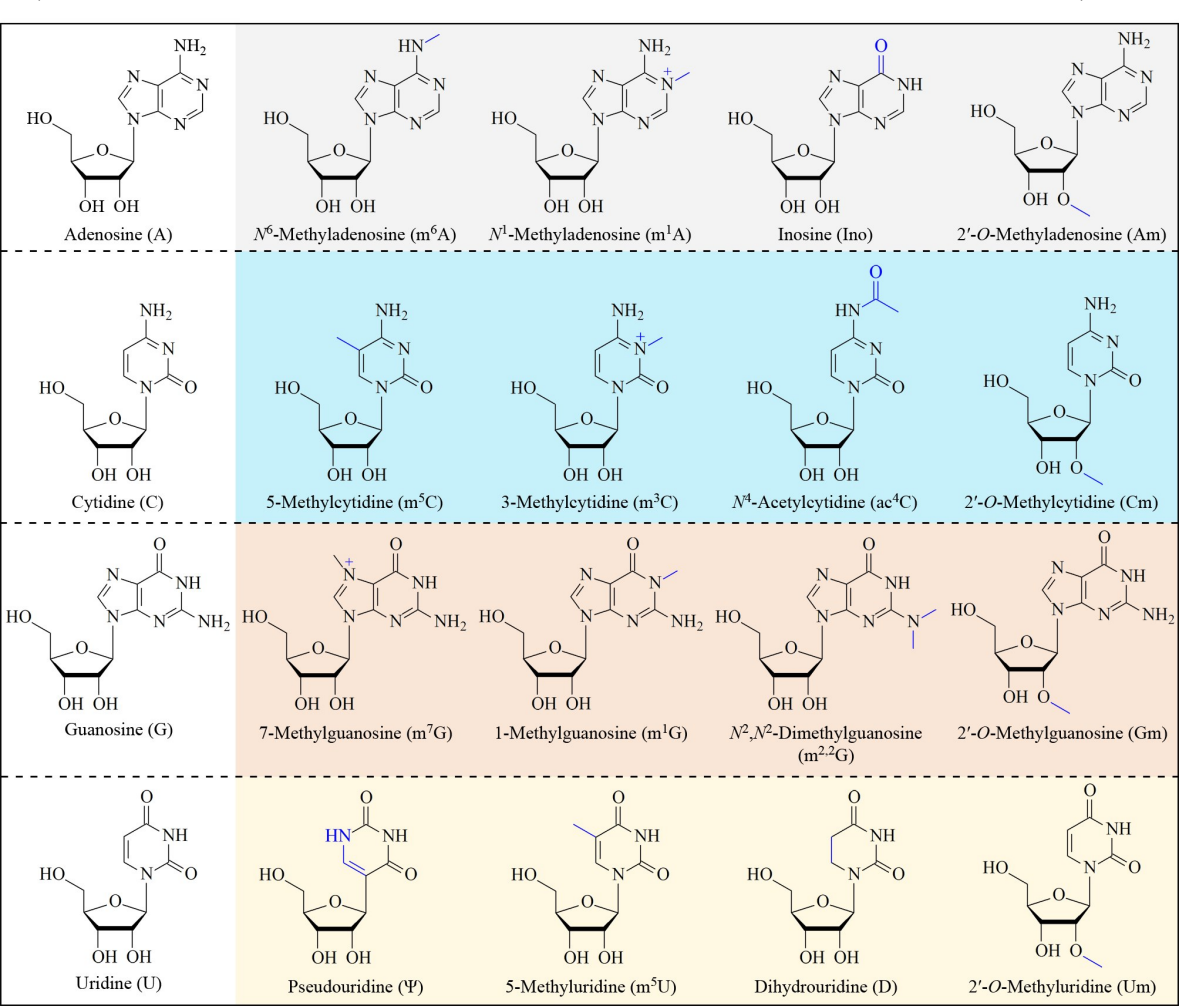
常见RNA修饰的化学结构

近年来,RNA修饰的测序定位技术取得了长足的进步^[69⇓-71]^,这些方法旨在解析RNA修饰位点、探索新的RNA修饰形式,是RNA修饰功能研究的重要工具。本文主要介绍了近年来基于测序技术的RNA修饰定位方法的进展,并对其发展前景予以展望。

## 1 现有的RNA修饰定位方法

RNA修饰与正常核苷的差异是实现其定位的基础,这些差异包括化学结构、生化反应活性及其生物合成途径等。根据前处理及测序方法的不同,现有的基于测序的RNA修饰定位技术可分为6类:直接高通量测序、抗体富集测序、酶辅助测序、化学辅助测序、代谢标记测序和纳米孔测序(见图2)。本文主要聚焦于这些方法的基本原理、优势和局限性。

**图2:**
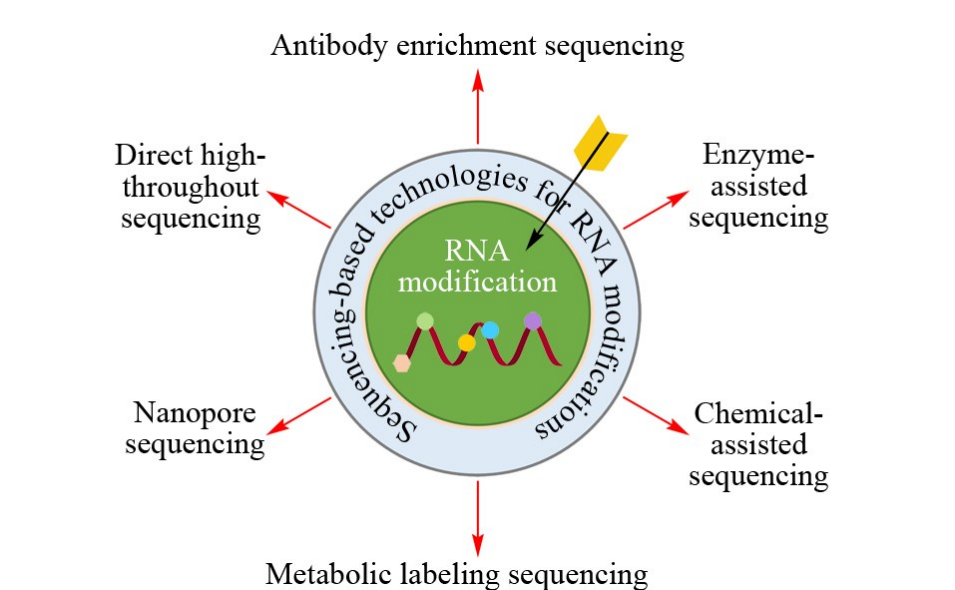
基于测序技术的RNA修饰定位分析方法示意图

### 1.1 直接高通量测序

逆转录过程中,某些RNA修饰能够破坏Watson-Crick碱基配对,导致逆转录突变,根据这一特征,利用高通量测序能够实现RNA修饰的定位。一个典型的例子是Ino,它是腺苷的水解脱氨产物,由RNA腺苷脱氨酶(ADAR)催化生成^[72]^。由于Ino的结构与鸟苷类似,它们都与胞嘧啶配对,在测序中读作G(原理见图3a)。基于此原理,研究人员结合高通量测序技术成功鉴定了人类转录组中数千个A到Ino的RNA编辑位点^[73⇓-75]^。然而,考虑到潜在的体细胞突变和单核苷酸多态性等因素,Ino的直接高通量测序方法假阳性较高^[76,77]^,需要额外的方法验证Ino的存在,并将其与体细胞突变或单核苷酸多态性导致的假阳性位点区分开来。

**图3:**
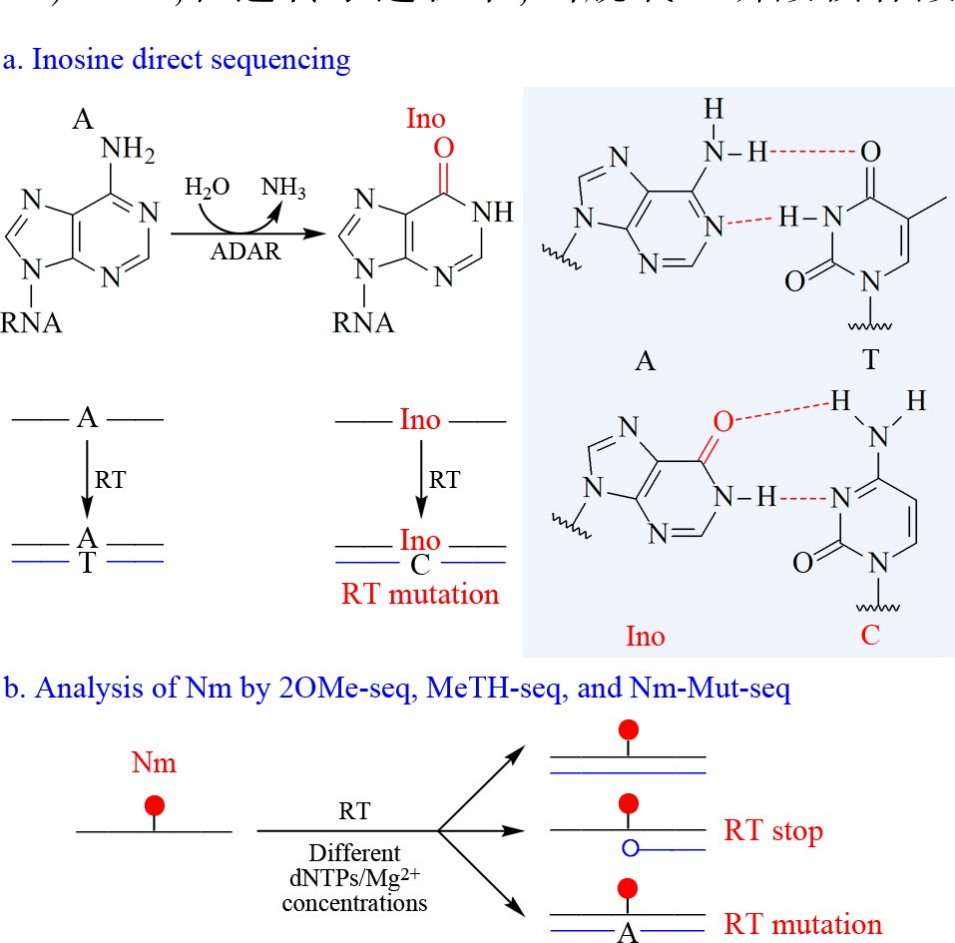
RNA修饰的直接测序技术方案

此外,通过优化逆转录条件,也能够造成逆转录终止或突变的特征,相关测序方法也被用于RNA修饰的定位分析。例如,2'-*O*-甲基化测序(2OMe-seq)和二羟基甲基化测序(MeTH-seq)方法能够以单碱基分辨率绘制Nm的全转录组图谱(原理见图3b)^[78,79]^,在逆转录过程中,当脱氧三磷酸核苷酸(dNTP)或Mg^2+^浓度降低时,Nm可阻断cDNA合成,通过识别端点处的碱基类型,能够在全转录组范围内准确地绘制Nm修饰图谱。最近,Chen等^[80]^开发了一种名为Nm突变测序(Nm-Mut-seq)的方法,该方法基于逆转录突变来绘制Nm图谱(原理见图3b)。在Nm-Mut-seq中,采用一种进化的逆转录酶,在其优化的限制性逆转录条件下,2'-*O*-甲基腺嘌呤(Am)、2'-*O*-甲基胞嘧啶(Cm)和2'-*O*-甲基鸟嘌呤(Gm)修饰位点发生逆转录突变(原理见图3b)。该方法即使在低丰度RNA中也能以单碱基分辨率检测Nm修饰。

总的来说,直接高通量测序的方法无需复杂的前处理流程,通过常规的RNA测序方法即可实现特定修饰的定位分析,且能够实现单碱基分辨率分析。然而,目前能够实现直接高通量测序的修饰种类较少,还需要开发针对其他类型RNA修饰的直接高通量测序方法。

### 1.2 抗体富集测序

大部分RNA修饰丰度较低,直接测序鉴定存在挑战,特别是对于RNA获取量较少的样品。由于特异性强,抗体富集后结合高通量测序已成为绘制RNA修饰图谱的重要方法。Dominissini等^[81]^提出了一种m^6^A-seq方法,该方法利用抗体捕获和高通量测序绘制了人和小鼠细胞RNA中的m^6^A。通过m^6^A-seq技术,他们在mRNA中鉴定了12000多个m^6^A峰,为m^6^A修饰的分布提供了全面的信息。另一种方法是由Meyer等^[82]^开发的甲基化RNA免疫沉淀测序(MeRIP-seq),他们将m^6^A特异性抗体富集与高通量测序相结合,实现了m^6^A的全转录组图谱的绘制。这种方法鉴定了哺乳动物mRNA中7676个含m^6^A的基因,揭示了哺乳动物体内m^6^A的动态调控。Schwartz等^[83]^还利用MeRIP-seq鉴定了酵母mRNA中的1308个m^6^A峰,并阐明了m^6^A在酵母mRNA中的调控作用。此外,研究还发现m^6^A在拟南芥mRNA中高度保守^[84]^,与哺乳动物类似,拟南芥mRNA中的m^6^A主要分布于终止密码子、3'-非翻译区(3'-UTR)和起始密码子附近。为了对少量RNA中的m^6^A测序,Chen等^[85]^建立了转座酶辅助的MeRIP-seq(tMeRIP-seq)方法,实现了不超过1000个细胞或60 ng总RNA中m^6^A的全转录组测序。

m^6^A-seq和MeRIP-seq方法的分辨率约为100~200个核苷酸,难以实现m^6^A位点的单碱基分辨率鉴定。为了解决这一难题,研究人员通过抗体-RNA之间的化学交联产生逆转录突变或终止信号,建立了m^6^A的单碱基分辨率定位方法,例如甲基化RNA交联和免疫沉淀(m^6^A-CLIP)^[86]^、m^6^A单核苷酸分辨率交联免疫沉淀(miCLIP)^[87,88]^和m^6^A水平和异构体鉴定测序(m^6^A-LAIC-seq)^[89]^。除了m^6^A, m^1^A也是各类RNA中广泛存在的修饰。利用m^1^A诱导逆转录突变或逆转录终止的特征,Li等^[90,91]^开发了基于m^1^A免疫沉淀的m^1^A免疫沉淀测序(m^1^A-ID-seq)和m^1^A错配辅助测序(m^1^A-MAP)方法,这些方法能够以单碱基分辨率绘制全转录组的m^1^A图谱。此外,Dominissini等^[56]^建立了m^1^A测序(m^1^A-seq),通过m^1^A抗体富集和m^1^A到m^6^A重排,鉴定了不同真核生物mRNA中的m^1^A。

抗体富集和高通量测序方法的整合使我们增进了对不同物种中RNA修饰及其调控功能的了解。然而,它们的分辨率仅限于大约100~200个核苷酸。虽然通过化学交联可以达到单碱基分辨率水平,但这些方法相对比较复杂。此外,这些方法中使用的抗体也存在特异性不足的问题,因此非靶向结合的问题亟待解决^[92]^。

### 1.3 酶辅助测序

利用与RNA修饰相关的酶,如甲基化酶、去甲基化酶、脱氨酶、核酸内切酶和核酸外切酶,研究者开发了相关的RNA修饰定位方法。m^6^Am是mRNA中一种常见的修饰,在mRNA的帽子结构附近的第一个转录核苷酸上具有高度保守性。帽子结构的甲基转移酶磷酸化CTD相互作用因子1(PCIF1)和去甲基化酶脂肪和肥胖相关蛋白(FTO)可动态调节m^6^Am的含量^[93,94]^。为了鉴定转录组中的m^6^Am和m^6^A, Sun等^[95]^利用m^6^Am去甲基化酶FTO开发了m^6^Am-seq。近期,Hu等^[96]^开发了m^6^A选择性烯丙基化学标记测序(m^6^A-SAC-seq),这是一种以单核苷酸分辨率定量绘制m^6^A全转录组图谱的方法(原理见图4a)。在m^6^A-SAC-seq中,他们利用了一种Dim1/KsgA家族的二甲基转移酶(MjDim1),这种酶能够将*S*-腺苷甲硫氨酸(SAM)的甲基加到m^6^A上,形成*N*^6^,*N*^6^-二甲基腺苷(*N*^6^,*N*^6^-dimethyladenosine, m^6,6^A)。作者使用SAM类似物(烯丙基-SAM),将m^6^A转化为烯丙基修饰的m^6^A,即*N*^6^-烯丙基,*N*^6^-甲基腺苷(*N*^6^-allyl, *N*^6^-methyladenosine, a^6^m^6^A)。随后通过I_2_处理将a^6^m^6^A转化为*N*^1^,*N*^6^-环化-m^6^A(*N*^1^,*N*^6^-cyclized-m^6^A),后者在cDNA合成过程中会诱导逆转录突变(见图4a)。m^6^A-SAC-seq方法只需约30 ng的mRNA或除掉rRNA的RNA,在较低起始RNA用量条件下实现了对m^6^A的定位分析。然而,MjDim1具有一定的序列偏好性,对于AAC序列中的m^6^A的反应性较差,在许多非m^6^A富集基序(DRACH)的序列上具有背景水平的反应活性,限制了对非甲基转移酶3/甲基转移酶14(METTL3/METTL14)甲基转移酶作用位点的研究。

**图4:**
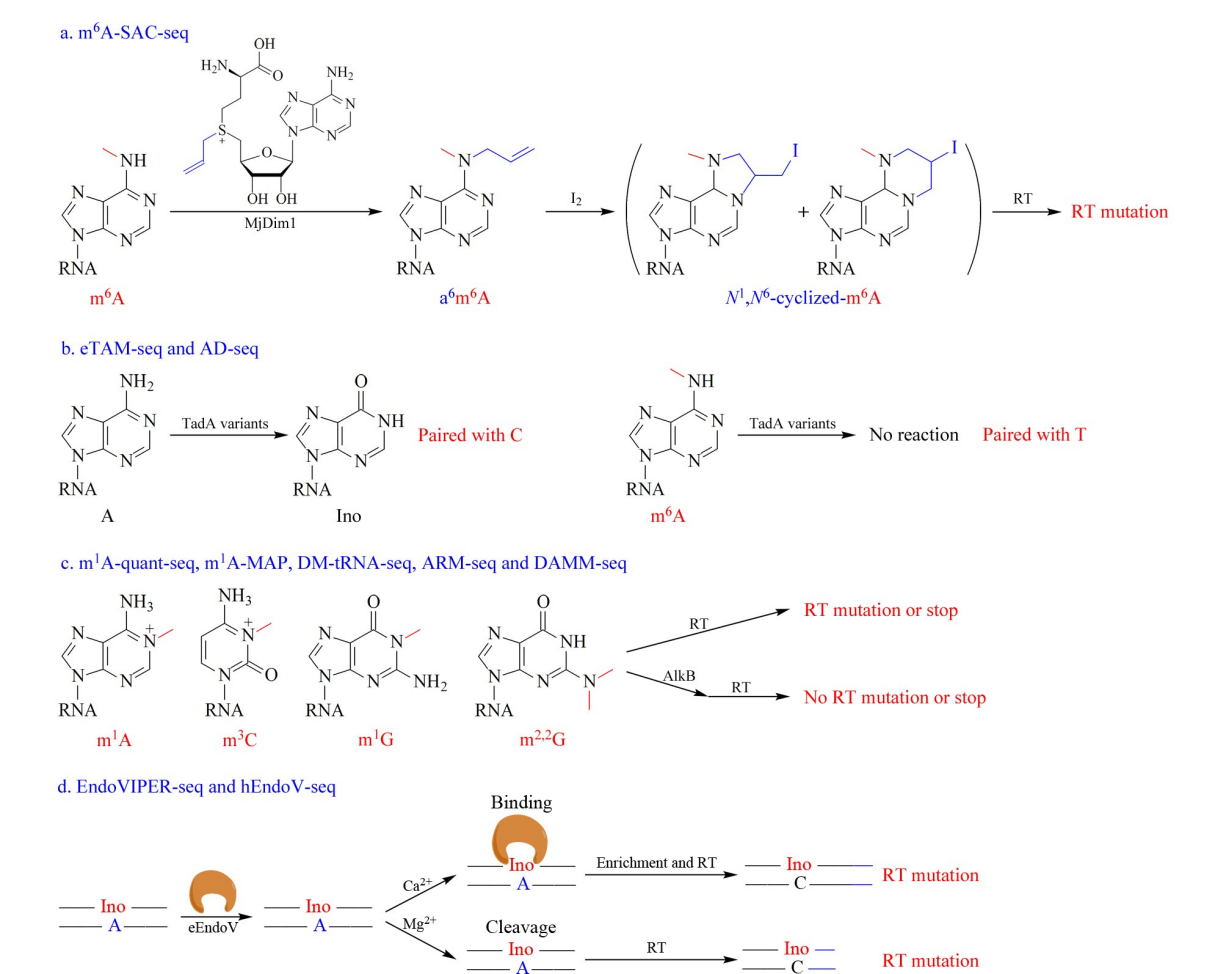
酶辅助测序技术用于RNA修饰定位分析的原理

此外,RNA脱氨酶也被用于绘制RNA修饰图谱。Meyer^[97]^开发了邻近RNA修饰靶向脱氨测序(DART-seq)方法,在DART-seq中,胞苷脱氨酶载脂蛋白B mRNA编辑酶催化亚基1(APOBEC1)与m^6^A结合蛋白的YTH结构域融合表达,诱导m^6^A残基邻近位点的C脱氨形成U。通过高通量测序分析C到T突变,可以确定m^6^A位点,该方法仅需10 ng总RNA即可实现m^6^A位点的准确鉴定。随后,作者还进一步开发了单细胞DART-seq(scDART-seq),实现了单细胞中m^6^A全转录组图谱的绘制^[98]^。由于无法直接定位m^6^A,这种方法并不能提供单碱基分辨率的m^6^A图谱。为了实现m^6^A的单碱基分辨率检测,Xiao等^[99]^和Shao等^[50]^开发了腺苷脱氨酶TadA辅助m^6^A测序(eTAM-seq)和腺苷脱氨测序(AD-seq)方法(原理见图4b)。在eTAM-seq和AD-seq中,大肠杆菌转移RNA腺苷脱氨酶(TadA)突变体TadA8.20、TadA8e或二聚体蛋白TadA-TadA8e能够选择性对A脱氨,而不作用于m^6^A。未甲基化的A被转化为Ino,后者与C配对,在测序过程中被识别为G。eTAM-seq和AD-seq方法实现了m^6^A的单位点深度测序及定量分析,即使只有10个细胞,也能完成对m^6^A的测序。这些方法依赖于脱氨酶的脱氨活性,脱氨酶的可及性是使用DART-seq、eTAM-seq和AD-seq检测m^6^A的先决条件,对于高度结构化的RNA,脱氨酶的脱氨活性受到抑制,限制了这些方法的应用。其他RNA修饰,如m^1^A、3-甲基胞嘧啶(3-methylcytidine, m^3^C)、1-甲基鸟苷(1-methylguanosine, m^1^G)和*N*^2^,*N*^2^-二甲基鸟苷(*N*^2^,*N*^2^-dimethylguanosine, m^2,2^G),由于修饰侧链基团位于Watson-Crick碱基配对区域,它们在cDNA合成过程中能够诱导逆转录终止或突变,利用这一特征,相关测序方法也已被开发出来^[54,90,100⇓⇓⇓-104]^(m^1^A-quant-seq、m^1^A-MAP、DM-tRNA-seq、ARM-seq和DAMM-seq,原理见图4c)。由于突变效率较低,这些方法在一些低丰度修饰的定位及定量检测上还存在困难。此外,在反转录过程中使用4SedTTP(原子特异性地在4位用硒取代氧)代替dTTP,可导致m^6^A位点逆转录的终止,因而实现了m^6^A和A的区分^[105]^。

核酸内切酶和外切酶在修饰和未修饰碱基之间的不同切割活性也被用于RNA修饰的测序(原理见图4d)。例如,大肠杆菌RNA核酸内切酶MazF能特异性识别并切割RNA中未甲基化的ACA基序,但不能切割m^6^ACA对应位点上的RNA^[106]^。利用这一特性,研究者提出了RNA核酸内切酶辅助测序方法(m^6^A-REF-seq和MAZTER-seq)^[107,108]^。尽管这两种方法能够实现m^6^A的单碱基分辨率检测,但是它们只能分析间隔较远的ACA基序中的m^6^A,而这些位点只占m^6^A位点的一小部分(16%~25%)。此外,据报道,内切酶V(EndoV)能在Mg^2+^存在下特异性切割含Ino修饰的RNA^[109]^。而用Ca^2+^取代Mg^2+^后,EndoV能够选择性结合Ino,但无法完成进一步的切割。利用这一特点,Knutson等^[110]^和Chen等^[111]^开发了基于EndoV的肌苷沉淀富集测序(EndoVIPER-seq和hEndoV-seq,原理见图4d)。此外,Koh等^[112]^还开发了m^6^A-交联-核酸外切酶测序(m6ACE-seq),用于以单碱基分辨率定量绘制m^6^A和m^6^Am的全转录组图谱。在m6ACE-seq中,首先将m^6^A抗体光交联到含m^6^A的RNA上,使其免受随后的5'至3'核酸外切酶XRN1消化的影响,随后将XRN1处理的结果与未经XRN1处理的结果进行比较,从而实现m^6^A和m^6^Am的定位。该方法依赖m^6^A抗体的特异性结合,抗体的选择性以及脱靶结合问题可能会对该方法造成挑战。

总之,考虑到酶促反应的特异性,酶辅助测序提高了RNA修饰核苷定位分析的准确性,实现了单碱基分辨率的定位和定量检测。然而,所用酶的反应活性、特异性和序列偏好性目前还存在不足,未来还需要对这些酶突变改进,从而获得活性更强、特异性更好、无序列偏差的突变蛋白。

### 1.4 化学辅助测序

由于转化率高、特异性好,化学标记已被广泛用于RNA修饰定位方法的开发。特定化学基团的引入,能够改变RNA修饰的逆转录特征,造成逆转录终止、突变或缺失。结合高通量测序,目前已开发出多种化学标记辅助的RNA修饰定位分析方法(见图5)。

**图5:**
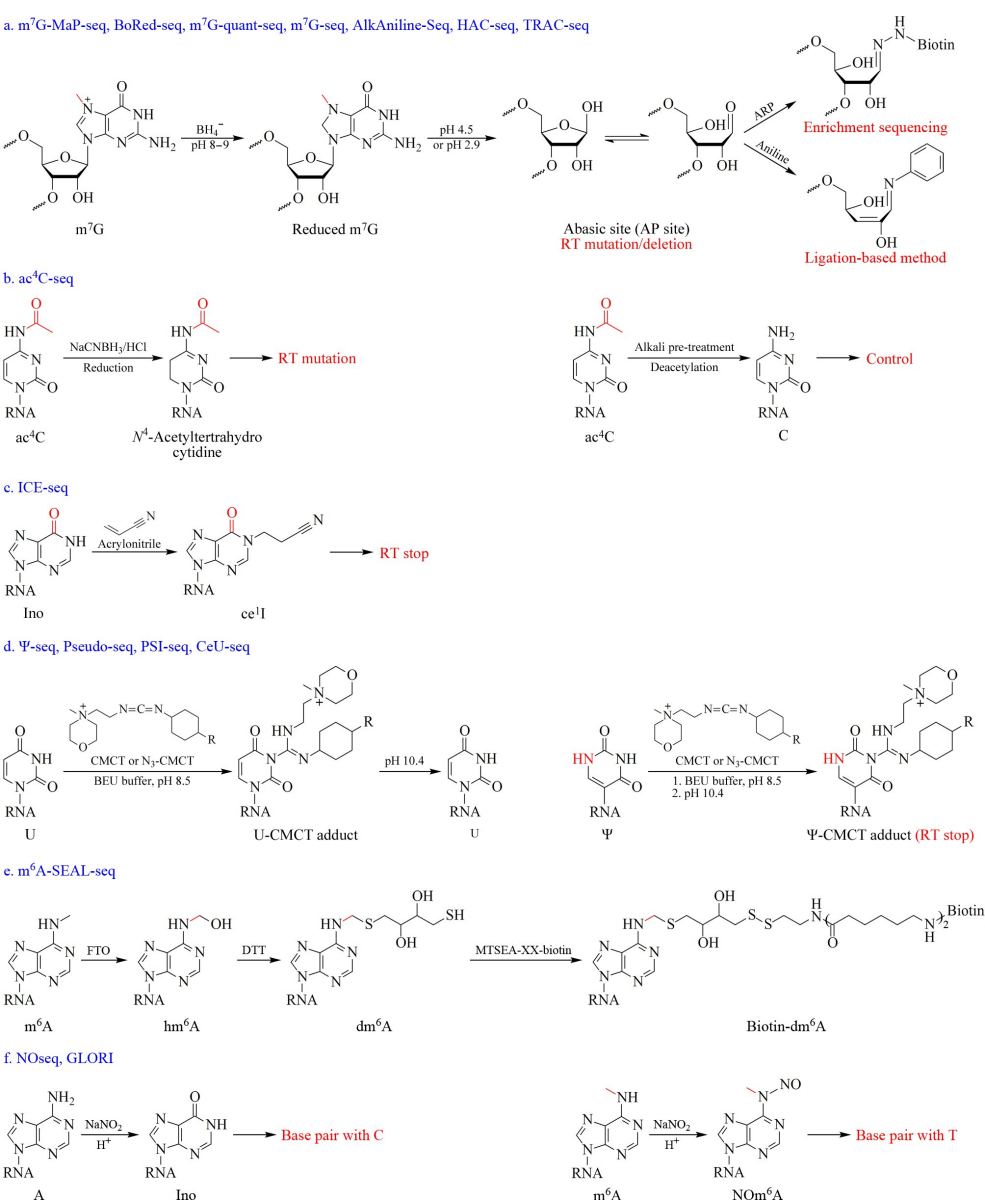
化学辅助测序技术用于RNA修饰定位分析的原理

某些RNA修饰,如m^7^G、ac^4^C、D,可以被硼氢化钠(NaBH_4_)或氰基硼氢化钠(NaCNBH_3_)还原^[113,114]^。例如,m^7^G能够被NaBH_4_还原转化为无碱基位点(AP site),在逆转录和测序过程中可检测到该位点的突变或缺失(原理见图5a)。基于此原理,已开发出多种方法用于检测rRNA、tRNA和miRNA中的m^7^G,包括m^7^G突变检测测序(m^7^G-MaP-seq)、硼氢化还原测序(BoRed-seq)和m^7^G定量测序(m^7^G-quant-seq)^[115⇓-117]^(原理见图5a)。为了对mRNA内部m^7^G进行测序,Zhang等^[57]^开发了m^7^G-seq方法,通过将含有生物素基团的醛基反应探针与m^7^G还原后形成的AP位点反应,从而实现含有m^7^G的mRNA的富集和测序(见图5a)。类似地,利用NaCNBH_3_还原的原理,Sas-Chen等^[118]^还开发了用于ac^4^C修饰定位的ac^4^C-seq方法(原理见图5b)。此外,通过碱水解将m^7^G和m^3^C还原为AP位点后,采用苯胺在AP位点处切断RNA,能够通过后续基于连接的方法实现m^7^G和m^3^C的检测,相关的方法包括AlkAniline-Seq^[119]^、肼苯胺裂解测序(HAC-seq)^[120]^和tRNA还原与裂解测序(TRAC-seq)^[121]^。利用这些方法,能够对tRNA和rRNA中的m^7^G和m^3^C进行单碱基分辨率检测(见图5a)。

与m^7^G和m^3^C修饰不同,D在碱性条件下与NaBH_4_反应后,嘧啶环打开后形成含有脲基团的产物^[122]^。在酸性条件下,该产物与带有伯氨基团的荧光团(如罗丹明)发生亲核取代反应,导致荧光团与脲基共价结合,这种超大荧光基团的引入能够导致逆转录终止^[123]^。基于此,有人开发了基于罗丹明标记的D修饰检测方法(Rho-seq和D-seq)^[124,125]^。通过此方法,实现了酵母tRNA、mRNA和snoRNA中的D修饰定位检测。

Nm是人类rRNA、tRNA和mRNA中一类常见的RNA修饰^[126]^,这些修饰在核糖体组装和翻译准确性方面起着至关重要的作用。为了对其进行测序,Birkedal等^[127]^开发了核糖甲基化测序(RiboMeth-seq)方法,该方法结合了RNA的碱水解和高通量测序,在碱性条件下,RNA随机断裂,但Nm会抑制水解。由于需要RNA的随机断裂,这种方法只局限于分析高丰度RNA(如rRNA)。为了克服这一局限,Dai等^[128]^开发了Nm-Seq。这种方法涉及RNA断裂和氧化-消除-磷酸化循环,通过去除3'至5'的2'-羟基核苷酸,暴露出3'端的内部Nm位点。Nm-Seq成功鉴定了哺乳动物mRNA中数千个2'-*O*-甲基化位点,发现Nm主要富集于编码序列(CDS)。基于类似的原理,研究者开发出了核糖氧化测序(RibOxi-seq)用于Nm测序分析^[129]^。此外,Zhang等^[130]^开发了Nm-REP-seq,该方法利用RNA核酸外切酶和高碘酸盐氧化反应去除2'-羟基核苷酸并结合测序来检测Nm位点。

为了克服直接高通量测序法检测Ino的局限性,Sakurai等^[131]^开发了一种名为“肌苷化学擦除测序(ICE-seq)”的方法,这种方法结合氰乙基化和反转录,实现了Ino的准确定位分析。在ICE-seq中,Ino与丙烯腈反应形成*N*^1^-氰乙基肌苷(*N*^1^-cyanoethinosine, ce^1^I)(原理见图5c)。ce^1^I的*N*^1^-氰乙基抑制了Watson-Crick碱基与C的互补配对,导致逆转录在ce^1^I处终止,通过测序可以确定Ino位点。作者利用ICE-seq在人类脑组织的mRNA中发现了5072个Ino位点,包括4395个新鉴定出来的RNA编辑位点。Ψ是最早被发现同时也是最丰富的RNA修饰之一^[69]^, Ψ可与*N*-环己基-*N*'-*β*-(4-甲基吗啉)乙基碳二亚胺(CMC)反应形成CMC-Ψ加合物^[132,133]^,后者能够造成逆转录终止,从而在随后的测序中以单碱基分辨率识别Ψ位点。基于此原理,目前已开发出以单碱基分辨率绘制Ψ位点的方法,包括Ψ-seq^[64]^、Pseudo-seq^[65]^和PSI-seq^[134]^(原理见图5d)。这些方法不能富集含Ψ的RNA片段,为了解决这一问题,Li等^[135]^开发了一种基于化学标记和生物素富集的Ψ测序(CeU-seq)方法,该方法通过在CMC中引入叠氮基团,从而通过生物素连接反应同时实现Ψ的特异性标记和富集(原理见图5d)。

亚硫酸氢盐测序(BS-seq)通常用于定位DNA中的5-甲基胞嘧啶(5-methylcytosine, 5mC),也被用于不同物种RNA中m^5^C的检测^[136⇓⇓⇓-140]^。与DNA类似,亚硫酸氢盐(bisulfite)处理会导致RNA中未修饰的C脱氨生成U,在逆转录过程中与A配对,而m^5^C不被亚硫酸氢盐脱氨,仍与G配对。Squires等^[137]^利用亚硫酸氢盐测序成功绘制了人类转录组中的m^5^C位点。为了在全转录组中同时检测m^5^C、Ψ和m^1^A, Khoddami等^[141]^开发了一种称为RBS-Seq的方法。在RBS-Seq中,m^1^A直接通过逆转录突变来鉴定,Ψ能够与亚硫酸氢盐反应形成单亚硫酸氢盐加合物,从而产生逆转录缺失特征。通过比较亚硫酸氢盐处理(BS)和非亚硫酸氢盐处理(NBS)的测序结果,能够同时识别RNA中的m^5^C、Ψ和m^1^A。此外,利用亚硫酸氢盐和Ψ的反应,Dai等^[142]^开发了亚硫酸氢盐诱导的缺失测序(BID-seq)。在BID-seq中,Ψ被定量转化为Ψ-BS加合物。这种方法只需10~20 ng的mRNA,即可实现人类细胞系和小鼠组织中Ψ位点的检测和定量。类似地,利用亚硫酸盐/亚硫酸氢盐混合物同时对Ψ处理的方式,Zhang等^[143]^开发了亚硫酸氢盐/亚硫酸盐处理分析假尿苷(PRAISE)方法,该方法通过增加亚硫酸氢盐反应过程中的有效离子浓度,抑制C脱氨,大大提高了Ψ的反应效率(>90%)。需要注意的是,亚硫酸氢盐处理的苛刻条件往往会导致RNA降解,从而使低丰度RNA中相关修饰检测困难。

为了克服m^6^A中甲基基团的化学反应惰性难题,Wang等^[144]^开发了一种FTO辅助的m^6^A选择性化学标记方法(m^6^A-SEAL-seq,原理见图5e)。在该方法中,m^6^A首先被去甲基化酶FTO氧化为*N*^6^-羟甲基腺苷(*N*^6^-hydroxymethyladenosine, hm^6^A),然后通过二硫苏糖醇介导的硫醇加成反应转化为更稳定的*N*^6^-二硫醇甲基腺苷(*N*^6^-dithiolsitolmethyladenosine, dm^6^A)。dm^6^A的自由巯基可与甲烷硫代磺酸(methanethiosulfonate, MTSEA)快速反应,实现在m^6^A上标记生物素,最终可被链霉亲和素珠富集并测序(见图5e)。此外,一氧化氮(NO)和亚硝酸钠(NaNO_2_)等化学物质的选择性氧化也可用于m^6^A测序(原理见图5f)^[145,146]^。Xie等^[147]^开发了一种基于NO/O_2_氧化的化学富集方法(m^6^A-ORL-seq),该方法利用NO/O_2_将m^6^A氧化为NOm^6^A,后者被二氧化硫脲(thiourea dioxide, TDO)还原成*N*^6^-氨基m^6^A(*N*^6^-amino m^6^A, Am^6^A),通过生物素探针标记,实现了m^6^A的富集与检测。然而,这些方法的特异性有限,在相同的反应条件下,G也可能被脱氨形成黄嘌呤核苷(X),后者也会造成逆转录突变。为了排除G反应产生的干扰,Liu等^[148]^利用乙二醛标记G,形成*N*^1^,*N*^2^-二羟基鸟苷加合物,亚硝酸钠氧化后,A转化为Ino, *N*^1^,*N*^2^-二羟基鸟苷加合物去保护,在碱性和加热条件下恢复为G,基于此,他们开发出了乙二醛和亚硝酸盐介导的未甲基化腺苷脱氨(GLORI)方法,该方法可对m^6^A进行精准定位分析(原理见图5f)。

化学辅助测序方法提供了一种无底物序列依赖性的RNA修饰检测策略,这些方法实现了多种RNA修饰的定位。此外,通过结合生物素亲和富集,实现了低丰度RNA修饰的富集和检测。尽管如此,很多化学反应的特异性还存在问题,未来还应继续开发特定修饰的特异性反应探针,实现RNA修饰的精准定位。

### 1.5 代谢标记测序

mRNA中m^6^A的生物合成涉及m^6^A甲基转移酶及其辅助因子SAM,除了天然底物SAM外,m^6^A甲基转移酶也能够利用SAM类似物进行生物合成反应。基于此,Shu等^[149]^开发了m^6^A-label-seq的方法用于单碱基分辨率检测mRNA中的m^6^A(原理见图6)。该方法采用烯丙基-L-硒代高半胱氨酸(allyl-SeAM)处理细胞,通过细胞内代谢将RNA中的m^6^A位点替换为*N*^6^-烯丙基腺苷(*N*^6^-allyladenosine, a^6^A), a^6^A能够通过I_2_诱导的环化反应转化为*N*^1^,*N*^6^-环化腺苷(Cyc-A),从而诱导逆转录突变(见图6)。

**图6:**
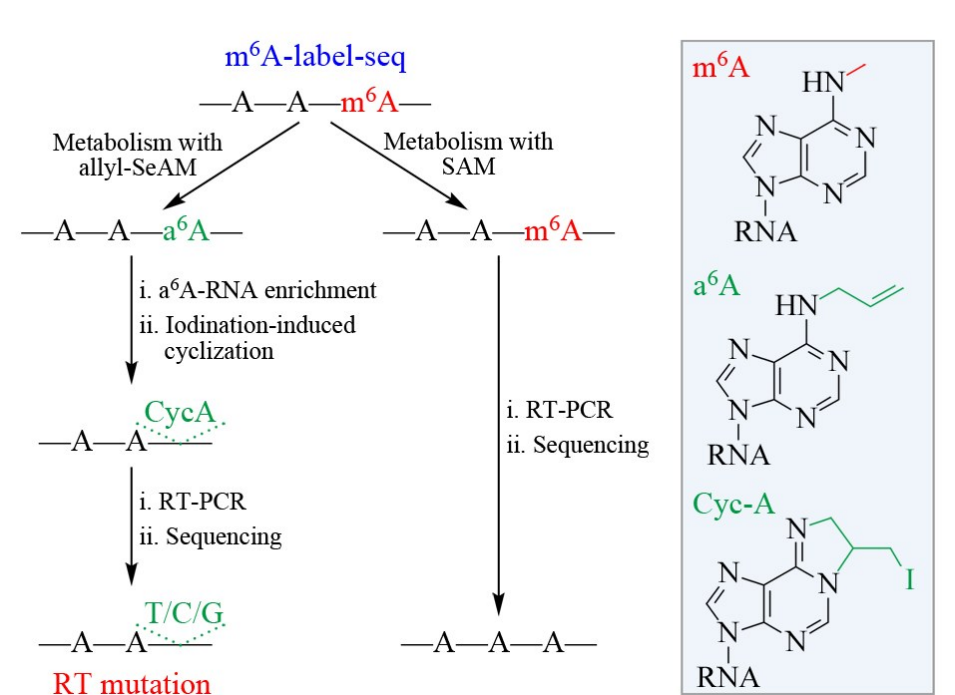
代谢标记测序技术m6A-label-seq的原理

此外,Chen等^[150]^开发了名为PA-m^6^A-seq的光交联辅助m^6^A测序,该方法采用4-硫代尿苷(4-thiouridine, s^4^U)处理细胞,s^4^U可被掺入到RNA分子中。通过免疫沉淀富集含m^6^A的mRNA,然后将s^4^U与m^6^A抗体进行紫外交联。共价交联的s^4^U在测序时被读作C,从而确定m^6^A位点。此外,Dai等^[151]^开发了5-FUrd-iCLIP-seq的方法,该方法利用5-氟尿苷(5-floxuridine, 5-FU)代谢掺入RNA中并与二氢尿苷合成酶DUS3L共价结合,结合后的蛋白被蛋白酶K水解,进而导致逆转录终止,基于此绘制出D修饰的图谱。此外,作者还利用5-乙炔基胞苷(5-ethynylcytidine, 5-EC)代谢标记和蛋白交联,开发了5-EC辅助的单核苷酸CLIP测序(5-EC-iCLIP-seq)和5-醛基胞苷(5-formylcytidine, f^5^C)的吡啶硼烷化学测序,用于分析tRNA上与5mC去甲基化酶ALKBH1相关的hm^5^C和f^5^C位点^[152]^。

作为一种RNA修饰的间接定位方法,代谢标记测序充分结合了RNA修饰及其相关合成酶,能够特异性定位修饰酶作用的位点,对于RNA修饰功能的研究具有重要意义。然而,该方法受限于RNA的细胞内标记,还无法应用于其他生物样本,如临床组织、血液等。

### 1.6 纳米孔测序

近年来,一种能够进行长阅读测序的第三代测序技术-纳米孔测序受到了广泛关注^[153]^。RNA分子通过蛋白质构成的纳米孔时,离子通过电流发生变化,由于修饰核苷与正常核苷离子通过电流的差异,能够实现多修饰的定位检测^[154]^。利用牛津纳米孔直接RNA测序(Oxford nanopore direct RNA sequencing)技术,Lorenz等^[155]^针对保守基序DRACH中的m^6^A位点构建了随机森林分类器,从而开发出基于纳米孔测序的m^6^A鉴定(MINES)方法,并采用MINES成功鉴定了HEK293T RNA中13000多个先前未发现的含有m^6^A修饰的DRACH位点。2020年,Kim等^[156]^报道了利用纳米孔直接RNA测序在SARS-CoV-2转录本上鉴定出41个潜在的RNA修饰位点。此外,Pratanwanich等^[157]^开发了基于纳米孔RNA测序的xPore方法,该方法实现了m^6^A位点的单碱基分辨率检测。2022年,Hendra等^[158]^推出了m^6^Anet,这是一种基于神经网络的方法,可通过一次纳米孔RNA测序在全转录组中鉴定和量化m^6^A。此外,纳米孔直接RNA测序已被用于鉴定人类、小鼠和爪蟾转录组中Ψ和Ino的位点^[159,160]^。最近还有研究者利用纳米孔直接RNA测序同时鉴定了tRNA、miRNA和rRNA中的多种修饰,如m^5^C、m^6^A、m^7^G、m^1^A、Ino、Ψ和D^[161⇓-163]^,基于纳米孔测序,Zhang等^[164]^建立了一个涵盖25个物种中16种RNA修饰的数据库(DirectRMDB)。

纳米孔测序提供了一种无需逆转录或PCR的直接RNA测序方法,然而,相比于二代测序技术,纳米孔测序的数据分析仍面临挑战,且需要解决假阳性率偏高的问题^[165,166]^。此外,纳米孔RNA测序需要大量RNA(约500 ng mRNA),不适合分析难以获得的样本。未来仍需努力改进纳米孔测序技术,开发更优异的纳米孔蛋白和强大的计算机算法。

## 2 总结与展望

分析技术的进步极大地推动了核酸表观遗传学领域的发展。为了全面了解RNA修饰的生物学功能,采用高灵敏度和高精确度的方法对其进行鉴定和定量至关重要。随着RNA修饰测序技术的进步,现在可以使用各种测序方法来分析特定的RNA修饰。不同方法之间也存在差异^[167,168]^,技术原理、抗体特异性、RNA测序偏差以及生物信息学分析方案等因素都可能影响最后的结果。对于绝大多数RNA修饰来说,研究人员须将所开发的新方法与现有方法进行比较验证。在比较不同方法的测序结果时,须谨慎考虑不同方法的原理、适用性和序列偏差。

本综述系统总结了高通量测序技术在RNA修饰中的应用,此外,作为一种高通量、高灵敏度的分析工具,液相色谱-质谱(LC-MS)也被用于一些rRNA和tRNA中修饰的定性和定位分析^[169⇓⇓⇓⇓⇓⇓⇓⇓-178]^。相比于高通量测序方法,LC-MS无需复杂的建库流程,操作简单、快速、通量高,具备准确的定性能力。然而,由于二级质谱难以区分,LC-MS方法在分析具有同分异构体的RNA修饰方面存在挑战;另外,对于序列信息更复杂、丰度更低的mRNA, LC-MS方法目前还难以实现特定位点修饰的准确定量。

全转录组范围内绘制RNA修饰图谱为理解RNA修饰在调控翻译和细胞过程中的作用提供了重要的证据。为了拓展对RNA表观转录组学领域的认识,须提高方法的适用性、准确性和可操作性。首先,要准确绘制RNA修饰图谱,关键是要开发不改变正常核苷酸的简单、直接的方法。目前的很多测序方法往往通过改变正常核苷,维持修饰核苷,这种策略需要提高对大量正常核苷的转化率,否则将产生假阳性结果。为了避开这种固有限制,开发仅转化修饰核苷的方法对于获得可靠而精确的图谱结果至关重要。其次,与DNA相比,RNA更容易降解。在新方法的开发过程中还应兼顾RNA的稳定性,确保在不损失RNA样品质量的情况下对RNA修饰进行准确定位。此外,非破坏性方法特别适用于较低RNA用量下修饰的定位分析,对于绘制如单细胞样品的RNA修饰图谱意义重大。第三,未来的测序技术还需应对在一次实验中对多种RNA修饰进行综合分析的挑战,从而更高效、全面地了解这些修饰的复杂调控网络。虽然已经报道了170多种RNA修饰,但只有部分修饰能够进行测序分析。为了全面了解RNA修饰的功能影响,未来的研究应着眼于开发针对这些修饰的测序方法,从而全面评估RNA表观转录组的生物学功能。
